# High population-attributable fractions of traditional risk factors for non-AIDS-defining diseases among people living with HIV in China: a cohort study

**DOI:** 10.1080/22221751.2021.1894904

**Published:** 2021-03-15

**Authors:** Jiaye Liu, Yuying Hou, Liqin Sun, Lifeng Wang, Yun He, Yang Zhou, Liumei Xu, Xiaoning Liu, Fang Zhao, Lukun Zhang, Hui Wang, Fu-Sheng Wang

**Affiliations:** a National Clinical Research Centre for Infectious Diseases, The Third People’s Hospital of Shenzhen and The Second Affiliated Hospital of Southern University of Science and Technology, Shenzhen, People’s Republic of China; b Medical School of Chinese PLA, Beijing, People’s Republic of China; c Treatment and Research Centre for Infectious Diseases, The Fifth Medical Centre of PLA General Hospital, Beijing, People’s Republic of China

**Keywords:** Human immunodeficiency virus, AIDS, incidence, population attributable fraction, non-AIDS-defining diseases

## Abstract

Morbidity and mortality of non-AIDS-defining diseases (NADs) have become the increasing burden of people living with HIV (PLWH) with long-term antiretroviral therapy (ART). We aimed to quantify the contribution of modifiable risk factors to NADs. We included PLWHs starting ART at the Third People’s Hospital of Shenzhen (China) from Jan 1, 2010 to Dec 31, 2017. We defined NAD outcomes of interest as cardiovascular disease (CVD), end-stage liver disease (ESLD), advanced renal disease (ARD), and non-AIDS-defining cancers (NADCs). We estimated incidence of outcomes and population-attributable fractions (PAFs) of modifiable traditional and HIV-related risk factors for each outcome. Overall, 8,301 participants (median age at study entry, 31 years) contributed 33,146 person-years of follow-up (PYFU). Incidence of CVD (362/100,000 PYFU) was the highest among outcomes, followed by that of ARD (270/100,000 PYFU), ESLD (213/100,000 PYFU), and NADC (152/100,000 PYFU). Totally, 34.14% of CVD was attributable to smoking, 7.98% to hypertension, and 6.44% to diabetes. For ESLD, 24.57% and 25.04% of it could be avoided if chronic hepatitis B and C virus infection, respectively, did not present. The leading PAFs for ARD were declined estimated glomerular filtration rate (eGFR) (39.68%) and low CD4 count (39.61%), followed by diabetes (10.19%). PAFs of hypertension, diabetes, and smoking for CVD, and declined eGFR and diabetes for ARD increased with age. The contribution of traditional risk factors for these NADs far outweighed the HIV-related risk factors. Individual-level interventions and population-level policy-making is needed to focus on these factors to prevent NADs in long-term management of HIV infection.

## Introduction

The incidences of AIDS-related events and death among people living with HIV (PLWH) have been dramatically reduced over the last two decades due to scale-up of HIV testing and antiretroviral therapy (ART) [1]. As a result, PLWH have longer life expectancy worldwide; however, non-AIDS-defining diseases (NADs), such as cardiovascular disease (CVD), non-AIDS-defining cancers (NADCs), liver and kidney diseases, were reported to be more prevalent in PLWH than HIV-uninfected individuals, which have become the main causes of death among PLWH in the era of modern ART [2,3].

The higher occurrences of those NADs among PLWHs are mediated by synergistic effect of traditional risk factors (including smoking, viral co-infection, dyslipidemia, diabetes, hypertension, etc.) [4,5] and HIV-related factors including immunodeficiency, inflammation, and hypercoagulability [6–8]. While several studies have reported the impact of these traditional and HIV-related factors on the progress of NADs through classical multivariate analysis, investigations to quantify the contribution of each individual factor to development of NADs seem not widely performed. Identification of the extent of the different factors that may contribute to the development of NAD is necessary for policymakers, programme implementers and public health experts to establish more targeted intervention strategies for NADs. Population-attributable fraction (PAF) reflects not only the risk of the outcome associated with the risk factor, but also the prevalence of the risk factor among PLWH with the outcome. The most important implication of PAF in clinic is to quantify the proportion of occurrence risk for NADs which could be reduced by modifying prevalent related factors or preventing exposing to them. By 2019, only one study from North America explored the contributions of modifiable risk factors to multiple NADs using PAF among PLWH [9]. Due to the different behavioural lifestyle characteristics, prevalence of risk factors, and HIV care policies among different PLWH cohorts, it is helpful and more informative to conduct these quantitative analyses in different settings. To the best of our knowledge, these quantitative analyses containing multiple NADs have not yet been conducted among PLWH in the rest of the world.

Therefore, we aimed to (1) estimate the incidences of major NADs based on a cohort of PLWH in China; (2) investigate the spectrum of comorbidity of more than one NAD; and (3) quantify the contribution of traditional and HIV-related risk factors to different NADs among PLWH by PAF.

## Methods

### Study design and participants

We conducted a retrospective cohort study at the Third People’s Hospital of Shenzhen (China), which is the only designated hospital for HIV care in Shenzhen. ART is provided free-of-charge in the hospital and ART regimen is prescribed according to the national HIV treatment guidelines. PLWH visited the hospital for continuous prescription of ART regimen as well as health monitoring with a predefined interval of three months. We included patients if they: (1) were ART-naïve and aged 15 years or older; and (2) entered care between January 1, 2010 and December 31, 2017. Patients without baseline information at entry and medical records were excluded from this study. Meanwhile, patients were also excluded if they had prevalent CVD, end-stage liver disease (ESLD), advanced renal disease (ARD) and NADC prior to, at, or within 3 months after, study enrolment (supplementary 1). We obtained baseline information and follow-up data, including socio-demographic, clinical information (exposures and outcomes), and laboratory test data from survey questionnaire, hospital information system and National Free Antiretroviral Treatment Program database [10].

The study protocol was compliant with the ethical guidelines of the 1975 Declaration of Helsinki and was approved by the Institutional Review Board of Shenzhen Third People’s Hospital. All participants provided written informed consent.

### Follow-up and outcomes

We assessed four outcomes: CVD, ESLD, ARD, and NADC. All of these NADs were ascertained by the diagnosis data (both inpatient and outpatient medical records), history data and related laboratory results. The composite of CVD included myocardial infarction, acute coronary syndrome, stroke, transient ischemic attack, and peripheral artery disease. ESLD was defined as having a clinical diagnosis of liver failure, compensated and decompensated liver cirrhosis, ascites, spontaneous bacterial peritonitis, variceal hemorrhage, hepatic encephalopathy, hepatocellular carcinoma, or evidence of laboratory value illustrating impaired hepatic function and hepatic fibrosis. To define ARD, we used diagnosis of chronic renal failure, severe renal insufficiency, uremia, end-stage renal disease, two consecutive estimated glomerular filtration rate (eGFR) < 45 mL/min/1.73 m^2^ at least 3 months apart, or having a history of hemodialysis or peritoneal dialysis, or renal transplantation. The NADC in this study was defined as the first diagnosed cancer, which would not include precancerous disease, Kaposi’s sarcoma, non-Hodgkin lymphoma, and cervical cancer.

Baseline was defined as the first time of hospital visit for ART for PLWH. We identified the outcomes occurring from three months after PLWH entry into the cohort to death, loss to follow-up, or Dec 31, 2019, the final date of the follow-up, whichever occurred first. The longest observation of patients was 10 years from study entry. Patients were regarded as loss to follow-up if they missed follow-up ≥90 days. The participants might develop two or more outcomes during the study period. While, for analysis of each specific outcome, the observation would be censored if the participants were diagnosed with the NAD analysed, lost to follow-up, or death.

### Exposure factors and covariates

Traditional risk factors included overweight (body mass index [BMI], 24≤ BMI <28 kg/m^2^) and obese (BMI ≥28 kg/m^2^) [11], smoking (ever vs. never), hypercholesterolemia (total cholesterol ≥5.2 mmol/L or prescription of lipid-modifying agents), hypertension (clinical diagnostic records or prescription of antihypertensive drugs), type 2 diabetes (clinical diagnostic records, prescription of diabetes medication, or a glycated hemoglobin ≥6.5%), declined eGFR (45≤ eGFR < 90 mL/min per 1.73 m² using the Chronic Kidney Disease Epidemiology Collaboration equation), alcohol consumption (reporting at least 1-month history of 5 drinks/week for women, or≥7 drinks/week for men), hepatitis B virus (HBV) infection (positive HBV surface-antigen test or positive envelope-antigen test, or a detectable HBV DNA), and hepatitis C virus (HCV) infection (positive anti-HCV antibody test or detectable HCV RNA). HIV-related factors included low CD4 count at initiation of ART (< 200 cells/µL), HIV viral suppression (plasma HIV-1 RNA < 500 copies/mL) at 3 months after ART initiation. Age and sex (male and female) were considered as non-modifiable covariates in this study. Individuals were categorized as having these modified factors or not on the basis of information of clinical diagnosis or history identified during the period mentioned above, and did not switch category during the observation period.

### Statistical analysis

We calculated the incidence for mono and multiple outcomes of interest by the number of events divided by the total person-years of follow-up (PYFU). We used Cox proportional hazards model to calculate adjusted hazard ratios (aHR) and corresponding 95% confidence intervals (CIs) for identifying factors associated with each of the outcomes. For participants developing two or more outcomes, we would include them repeatedly for estimating the incidence for different outcome in the Cox model. The calculation of PAF was based on the proportion of individuals with the risk factor among all participants and factors associated with each outcome using the results of Cox model. The approach of PAFs used in this study was illustrated by Laaksonen et al. [12], which is suitable for cohort study. Since the risk factors that were related to the incidence of the disease of interest were also related to mortality, the modification of these risk factors would affect both the risk of disease outcomes and the risk of death. Thus, competing risk due to death was also taken into account in the estimation for outcomes in this study. The PAF estimation for NADC was expected, however, it was not actually done as lack of convergence on the process of model estimation of PROC LIFERREG. Subgroup analyses were conducted for the CVD, ESLD and ARD among PLWHs stratified by age (15–29, 30–49, and ≥50 years old) and sex. *P* values < 0.05 and PAF 95% CIs not crossing 0% were deemed statistically significant. All analyses were conducted using SAS software version 9.4 (SAS Institute).

## Results

### Demographic and clinical characteristics

From Jan 1, 2010 to Dec 31, 2017, 9,180 PLWHs sought HIV care at this hospital. After excluding 95 individuals with prevalent NADs, 20 aged less than 15 years old, 764 lacking baseline or follow-up records, 8,301 (90.4%) participants were included in our analyses (supplementary 1). Of 8,301 participants, 7,444 (89.7%) were male and the median age was 31 years old (IQR 26, 39); and 5,354 (64.5%) acquired HIV via male-to-male sex contact. The median follow-up duration of the participants included to assess the four outcomes was 3.7 years (IQR 2.6–4.9).

### Incidence of NADs among participants

**Figure 1.**
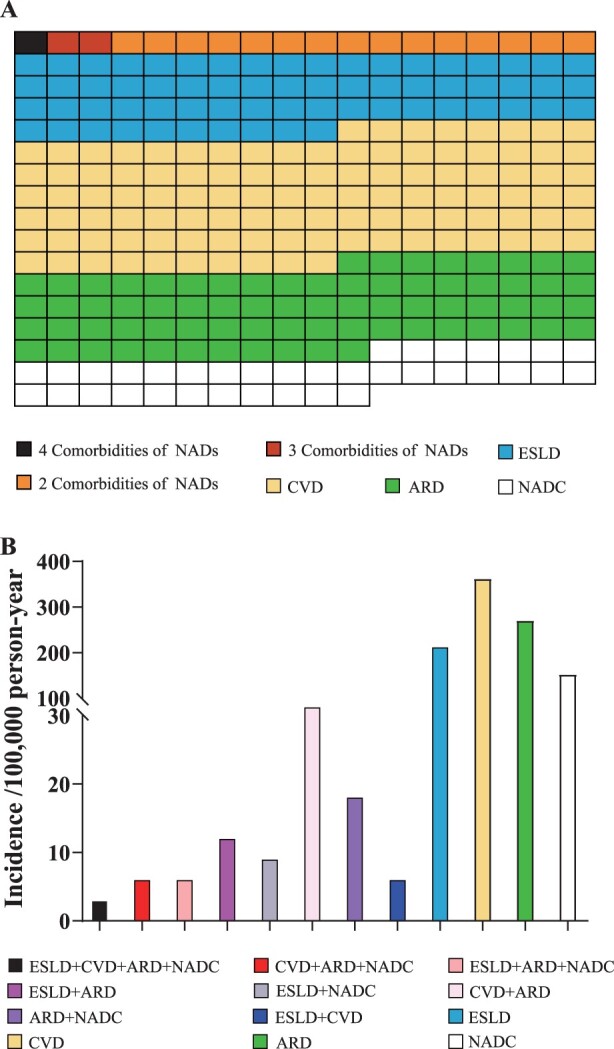
Incident non-AIDS-defining diseases. Cross-section of number of patients with different comorbidities of non-AIDS-defining diseases between 2010 and 2019, based on observed 299 patients who developed non-AIDS-defining diseases (each square represents a patient) (A). Incidences of various comorbidities of non-AIDS-defining diseases between 2010 and 2019 (B). ARD: Advanced renal disease. CVD: cardiovascular disease. ESLD: end-stage liver disease. NADC: non-AIDS-defining cancer. NADs: non-AIDS-defining diseases.

During a total of 33,146 PYFU, 299 participants were diagnosed with at least one of the four NADs: 119 participants with CVD, 89 ARD, 70 ESLD, and 50 NADC (supplementary 1). Moreover, 15 participants developed two NADs and 3 developed three or four NADs. (Figure 1(A)). The incidence of CVD of the cohort was 362 per 100,000 PYFU, which was higher than that of ARD (270 per 100,000 PYFU), ESLD (213 per 100,000 PYFU), and NADC (152 per 100,000 PYFU). Of the participants with at least two NADs, co-morbidities of CVD and ARD had the highest incidence (30 per 100,000 PYFU) (Figure 1(B)).

### The prevalence of modifiable factors in participants with or without NADs

Compared with those who did not develop the outcomes, participants with any of the four incident outcomes were more likely to be aged ≥30 years old, have acquired HIV infection via heterosexual contact, and have a history of smoking and CD4 count <200 cells/μL at the initiation of ART, and were less likely to be male, and have a history of alcohol use (Table 1). Of note, participants with a diagnosis of CVD or ARD had a higher prevalence of hypertension, diabetes, declined eGFR and hypercholesterolemia, while participants with a diagnosis of ESLD had a significantly higher prevalence of HBV or HCV infection.

**Table 1.** Demographic characteristics and prevalence of modifiable factors in participants with or without NADs.

	Total	ESLD		CVD		AKD		NADC	
		No diagnosis	Diagnosis	No diagnosis	Diagnosis	No diagnosis	Diagnosis	No diagnosis	Diagnosis
**Demographics**	8301	8231	70	8182	119	8212	89	8251	50
Age group									
<30 years	3541 (42.7)	3523 (42.8)	18 (25.7)	3528 (43.1)	13 (10.9)	3539 (43.1)	2 (2.3)	3533 (42.8)	8 (16.0)
30–49 years	4171 (50.3)	4125 (50.1)	46 (65.7)	4106 (50.2)	65 (54.6)	4133 (50.3)	38 (42.7)	4144 (50.2)	27 (54.0)
≥50 years	589 (7.0)	583 (7.1)	6 (8.6)	548 (6.7)	41 (34.5)	540 (6.6)	49 (55.0)	574 (7.0)	15 (30.0)
Male	7444 (89.7)	7386 (89.7)	58 (82.8)	7342 (89.7)	102 (85.7)	7376 (89.8)	68 (76.4)	7402 (89.7)	42 (84.0)
HIV transmission route									
Male-to-male sex contact	5354 (64.5)	5325 (64.7)	29 (41.4)	5306 (64.9)	48 (40.3)	5325 (64.9)	29 (32.6)	5333 (64.6)	21 (42.0)
Heterosexual contact	2714 (32.7)	2685 (32.6)	29 (41.4)	2651 (32.4)	63 (52.9)	2664 (32.4)	50 (56.2)	2688 (32.6)	26 (52.0)
IDU	77 (1.0)	70 (0.9)	7 (10.0)	75 (0.9)	2 (1.7)	74 (0.9)	3 (3.4)	74 (0.9)	3 (6.0)
Other	156 (1.8)	151 (1.8)	5 (7.2)	150 (1.8)	6 (5.0)	149 (1.8)	7 (7.9)	156 (1.9)	0 (0.0)
**Traditional risk factors**									
Smoking	1946 (23.4)	1923 (23.4)	23 (32.9)	1883 (23.0)	63 (52.9)	1913 (23.3)	33 (37.1)	1932 (23.4)	14 (28.0)
Alcohol consumption	1916 (23.1)	1905 (23.1)	11 (15.7)	1902 (23.3)	14 (11.8)	1901 (23.2)	15 (16.9)	1909 (23.1)	7 (14.0)
BMI (kg/m^2^)									
<18.5	1232 (14.8)	1219 (14.8)	13 (18.6)	1218 (14.9)	14 (11.8)	1222 (14.9)	10 (11.2)	1226 (14.9)	6 (12.0)
18.5–23.9	5760 (69.4)	5713 (69.4)	47 (67.1)	5677 (69.4)	83 (69.8)	5698 (69.4)	62 (69.7)	5729 (69.4)	31 (62.0)
≥24	1309 (15.8)	1299 (15.8)	10 (14.3)	1287 (15.7)	22 (18.5)	1292 (15.7)	17 (19.1)	1296 (15.7)	13 (26.0)
Hypertension	124 (1.5)	123 (1.5)	1 (1.4)	108 (1.3)	16 (13.5)	116 (1.4)	8 (9.0)	122 (1.5)	2 (4.0)
Diabetes	94 (1.1)	91 (1.1)	3 (4.3)	79 (1.0)	15 (12.6)	79 (1.0)	15 (16.9)	93 (1.13)	1 (2.0)
Hypercholesterolemia	103 1(12.4)	1020 (12.4)	11 (15.7)	1010 (12.3)	21 (17.7)	1015 (12.4)	16 (18.0)	1025 (12.4)	6 (12.0)
Declined eGFR	485 (5.8)	481 (5.8)	4 (5.7)	470 (5.7)	15 (12.6)	435 (5.3)	50 (56.2)	475 (5.8)	10 (20.0)
HBV infection	939 (11.3)	913 (11.1)	26 (37.1)	925 (11.3)	14 (11.8)	932 (11.4)	7 (7.9)	931 (11.3)	8 (16.0)
HCV infection	168 (2.0)	150 (1.8)	18 (25.7)	164 (2.0)	4 (3.4)	166 (2.0)	2 (2.3)	166 (2.0)	2 (4.0)
**HIV-related risk factors**									
CD4 count <200 cells/μL	3056 (36.8)	3020 (36.7)	36 (51.4)	2997 (36.6)	59 (49.6)	2990 (36.4)	66 (74.2)	3031 (36.7)	25 (50.0)
HIV RNA <500 copies/ml at 3 months after ART initiation	7096 (85.5)	7032 (85.4)	64 (91.4)	6998 (85.5)	98 (82.4)	7028 (85.6)	68 (76.4)	7052 (85.5)	44 (88.0)
ART regimen									
3TC + TDF + EFV/NVP	6230 (75.0)	6189 (75.2)	41 (58.6)	6173 (75.4)	57 (47.9)	6209 (75.6)	21 (23.6)	6197 (75.1)	33 (66.0)
3TC + AZT/D4T + NVP/EFV	1034 (12.5)	1026 (12.4)	8 (11.4)	1014 (12.4)	20 (16.8)	1022 (12.4)	12 (13.5)	1026 (12.4)	8 (16.0)
3TC + LPVr + TDF/AZT/ D4T	699 (8.4)	689 (8.4)	10 (14.3)	669 (8.2)	30 (25.2)	681 (8.3)	18 (20.3)	692 (8.4)	7 (14.0)
Other	338 (4.1)	327 (4.0)	11 (15.7)	326 (4.0)	12 (10.1)	300 (3.7)	38 (42.7)	336 (4.1)	2 (4.0)

Note: Data are presented as n (%). ART: antiretroviral therapy; BMI: body-mass index; eGFR: estimated glomerular filtration rate; HBV: hepatitis B virus; HCV: hepatitis C virus; IDU: injection drug use; INSTIs: integrase inhibitors; NADs: non-AIDS-defining diseases; TDF: tenofovir disoproxil fumarate; 3TC: lamivudine; D4T: stavudine; EFV: efavirenz; NVP: nevirapine; AZT: zidovudine; LPVr: lopinavir/ritonavir.

### PAF calculation for NADs of interest

**Figure 2.**
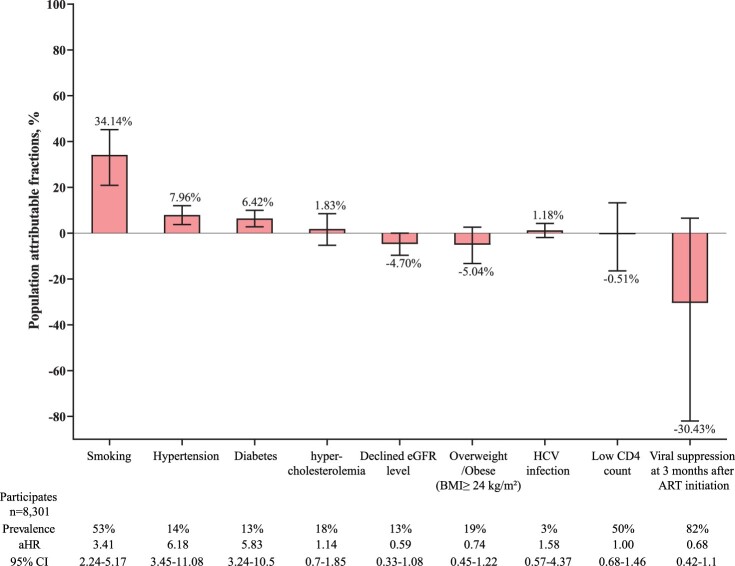
Population attributable fractions for traditional and HIV-related risk factors for cardiovascular diseases. Whiskers indicate 95% CI. Below the plot, prevalence is the prevalence of the risk factor at study entry among those with incident cardiovascular diseases. aHRs were adjusted for age and sex. aHR: adjusted hazard ratio. ART: antiretroviral therapy. eGFR: estimated glomerular filtration rate. BMI: body-mass index. HCV: hepatitis C virus.

In the PAF analyses, 34.14% (95% CI 20.89–45.17%) of the occurrences of CVD were attributable to smoking, 7.96% (95% CI 3.78–11.98%) were attributable to hypertension, and 6.42% (95% CI 2.75–9.97%) were attributable to diabetes (Figure 2). In subgroup analyses stratified by age, patients older than 50 years old presented with the highest PAF of smoking (40.17%, 95% CI 25.83–51.74%). The PAF of hypertension for CVD increased with age (2.29% in < 30-year-old vs. 5.51% in 30–49-year-old vs. 15.82% in ≥50-year-old; pairwise comparisons, all *P* < 0.01), and a similar trend was also observed in term of PAF of diabetes (0.92% in <30-year-old vs. 4.39% in 30–49-year-old vs. 13.35% in ≥50-year-old; pairwise comparisons, all *P* < 0.01) (supplementary 2). The PAF of smoking was much higher in male participants than that in female participants (38.07% vs. 8.13%; *P* < 0.01) (supplementary 3).

**Figure 3.**
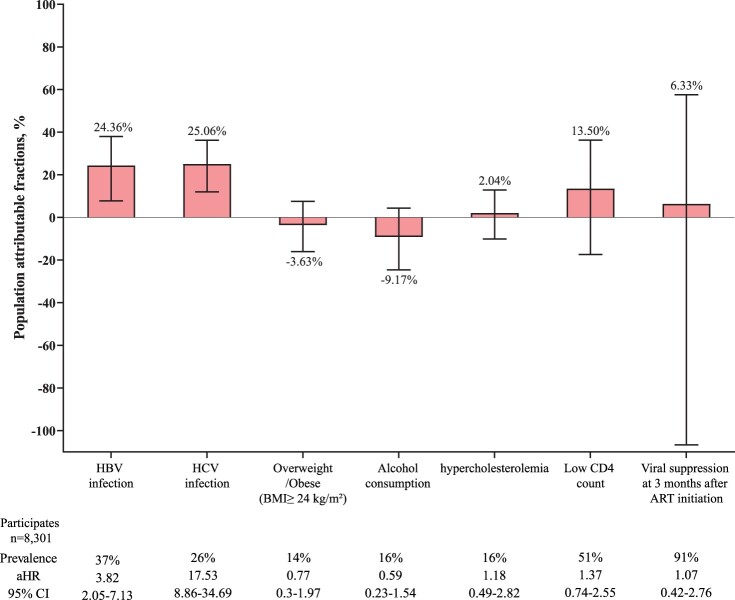
Population attributable fractions for traditional and HIV-related risk factors for end-stage liver disease. Whiskers indicate 95% CI. Below the plot, prevalence is the prevalence of the risk factor at study entry among those with incident end-stage liver diseases. aHRs were adjusted for age and sex. aHR: adjusted hazard ratio. ART: antiretroviral therapy. BMI: body-mass index. HBV: hepatitis B virus. HCV: hepatitis C virus.

The PAF of HBV infection and HCV infection for ESLD was 24.36% (95% CI 7.76–37.97%) and 25.06% (95% CI 11.99–36.19%), respectively (Figure 3). In the subgroup analyses stratified by age, the PAF of HBV infection and that of HCV infection for ESLD among participants aged 30–49 years old were significantly higher than those among other age groups (supplementary 4). The PAF of HBV infection was similar between male and female participants (24.82% vs. 21.60%), while the PAF of HCV infection was significantly higher in female participants (38.91% vs. 22.76%; *P* < 0.001) (supplementary 5).

**Figure 4.**
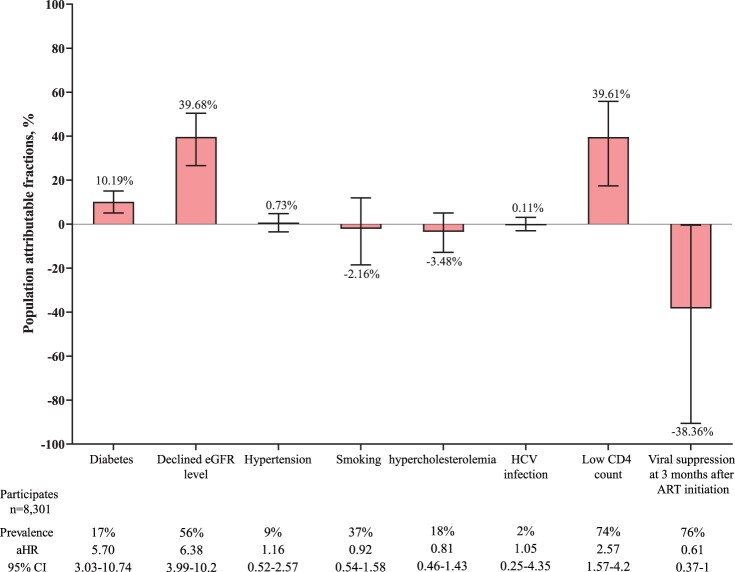
Population attributable fractions for traditional and HIV-related risk factors for advanced renal disease. Whiskers indicate 95% CI. Below the plot, prevalence is the prevalence of the risk factor at study entry among those with incident advanced renal diseases. aHRs were adjusted for age and sex. aHR: adjusted hazard ratio. ART: antiretroviral therapy. eGFR: estimated glomerular filtration rate. HCV: hepatitis C virus.

The leading PAFs for ARD in PLWHs were declined eGFR (39.68%, 95% CI 17.41–55.83%) and CD4 count<200 cells/µL (39.61%, 95% CI 26.59–50.43%), which were higher than that of diabetes (10.19%, 95% CI 5.04–15.05%) (Figure 4). Both PAFs that could be attributed to declined eGFR (7.39% in <30 year-old vs. 26.83% in 30–49 year-old vs. 52.19% in ≥50 year-old; pairwise comparisons, all *P* < 0·001) and diabetes (0.83% in <30 year-old vs. 4.00% in 30–49 year-old vs. 15.91% in ≥50 year-old; pairwise comparisons, all *P* < 0.01) increased across age groups, while PAF of low CD4 cell count was the highest in patients aged 30–49 years old (supplementary 6). The PAF of declined eGFR was much higher in male participants than that in the female (41.21% vs. 34.29%; *P* = 0.003), and a similar result was also observed in term of PAF of diabetes (11.24% vs. 6.47%; *P* = 0.004) (supplementary 7).

## Discussion

In this cohort of ART-naïve PLWH in China during 2010–2019, we estimated that the incidence of CVD, ARD, ESLD and NADC was 362, 270, 213, and 152 per 100,000 PYFU, respectively, after initiation of ART. Traditional risk factors contributed significantly to the development of these outcomes of interest: smoking, hypertension, and diabetes for CVD, HBV and HCV infection for ESLD, and declined eGFR and diabetes for ARD. The findings of this study can inform the programmes and policies for preserving health among PLWH.

Based on this long-term follow-up cohort, we found that CVD had become the leading NAD among PLWH receiving ART. Being consistent with our results, a modelling study showed that 78% of PLWH would progress to CVD in 2030 in the Netherlands cohort [13]. In addition, a systematic review that included 80 longitudinal studies, containing 793,635 HIV-infected patients, concluded that the risk of stroke and myocardial infarction among PLWH was increased by 2.16 (95% CI 1.68–2.77) compared with that in the uninfected population [14]. In our study, the incidence of CVD was higher than that reported in general population with similar age to our cohort [15]. These data imply that CVD will become the leading burden among the NADs in PLWH.

Given the higher morbidity of CVD and associated disease burden, it is a priority to identify the leading risk factors of CVD and implement health interventions among PLWH. In this study, we found that over one third of the occurrences of CVD in our participants were attributable to smoking, which was consistent with the findings of a previous study [9]. Although stopping smoking has been confirmed beneficial and strongly recommended in clinical practice, smoking cessation remains a challenge for PLWH in various areas [16–18]. The PAF of smoking to CVD in our study shows more straightforward information for PLWH to recognize quantified benefit from smoking cessation and provides supporting data for physicians to implement behavioural and adherence counselling. In addition, hypertension and diabetes also presented significant associations with this outcome, and their contribution increased with age in our study. Hence, it is important to evaluate all these factors of PLWH when they are successfully linked to HIV care, where traditional factors are more likely to be neglected in clinical practice. It has been reported that the proportion of PLWH not knowing their elevated blood pressure was approximately two times higher than that of general population [19]. In this study, we did not find an association between hypercholesteremia and CVD, despite the fact that participants with CVD had higher prevalence of hypercholesteremia. With total cholesterol ≥7·2 mmol/L considered as an independent high-risk factor for CVD [20], only 40 patients in our study had this high level of total cholesterol, which limited our analysis to identify the association.

We estimated that both the fractions of preventable ESLD attributable to HBV infection and HCV infection were 25%. The significant contribution of these two factors for ESLD were also identified in the HIV cohort from North America [9]. Our previous study showed that the seroprevalence of HBV among our PLWH was 10.46% [21], which was obviously higher than that (6.1%) in general population in China [22]. Given the endemic situation of HBV infection and higher prevalence of co-infection with HBV among PLWH in China, our study highlights that routine screening of HBV infection for all PLWHs seeking HIV care, HBV vaccination for PLWHs without protective antibodies, and use of ART containing antiviral-drugs to HBV for co-infected patients are essential. Although only 2% of our participants were coinfected with HCV in this study, the high prevalence of HCV infection in participants with ESLD resulted in a similar fraction to HBV infection. The availability of direct-acting antiviral agents for HCV infection with high cure rates makes elimination of this risk factor for ESLD achievable. Thus, early detection of HCV infection should be also strengthened, especially at the time when PLWH enter HIV care.

It has been reported treatment-naïve PLWH had a higher prevalence of renal dysfunction [23,24]. Thus, we selected declined eGFR at baseline as a modifiable traditional factor in this study, and found almost 40% of the ARD could be attributed to declined eGFR level. This result suggests it is necessary to evaluate the renal function at the time of entry into HIV care. Close monitoring and timely intervention, and avoiding using nephrotoxic ART are beneficial to reduce the risk of ARD. In addition, lower CD4 counts showed a significant association with occurrence of ARD. Although early ART initiation was widely scaled-up, more than 40% of PLWHs entered care with CD4 counts < 200 cells/μL in China [25]. More efforts are needed to improve access to HIV care and early ART initiation, which is important not only to promote immune recovery but to reduce the risk of ARD.

There are several limitations of our study. First, we did not estimate the PAF attributed to antiretroviral regimens. In clinical practice, antiretroviral regimens were prescribed in consideration of indications. Thus, to investigate the PAF for antiretroviral regimens will be confounded by indication; moreover, interactions between antiretroviral regimens and risk factors are needed to be taken into account. In addition, regimen changes were frequent due to adverse events and drug–drug interaction, which might result in a high proportion of participants to be censored at the time of regimen change in the estimation of PAF for specific antiretroviral regimens. Second, the prevalence of hypertension was probably underestimated as the hypertension was determined by diagnosis or having antihypertensive drugs in this study. Third, the prevalence of HCV infection might be overestimated as HCV infection were diagnosed based on a positive anti-HCV antibody. This overestimation might dilute the association between HCV infection and the outcomes. Fourth, the median follow-up time (3.7 years) remained short, and some NADs may need a longer observation duration to develop.

In summary, our study provides new population-based estimate of the incidences of NADs among PLWH in China. Smoking, hypertension and diabetes were the leading factors contributory to CVD among PLWH, HBV and HCV infection to ESLD, and declined eGFR level, low CD4 cell count at initiation of ART, and diabetes to ARD. Traditional risk factors accounted for larger shares of the causation for NADs. Individual-level interventions and population-level policy-making are needed to focus on modifiable traditional factors to prevent NADs among PLWH who are successfully treated with ART.

## Supplementary Material

Supplement.doc
