# Supplementary material for: High population-attributable fractions of traditional risk factors for non-AIDS-defining diseases among people living with HIV in China: a cohort study
Source: Emerg Microbes Infect. 2021 Mar 15;10(1):416–23. doi: 10.1080/22221751.2021.1894904 (PMC7971336; doi:10.1080/22221751.2021.1894904)
Supplement: Supplement.doc [file TEMI_A_1894904_SM0278.doc]

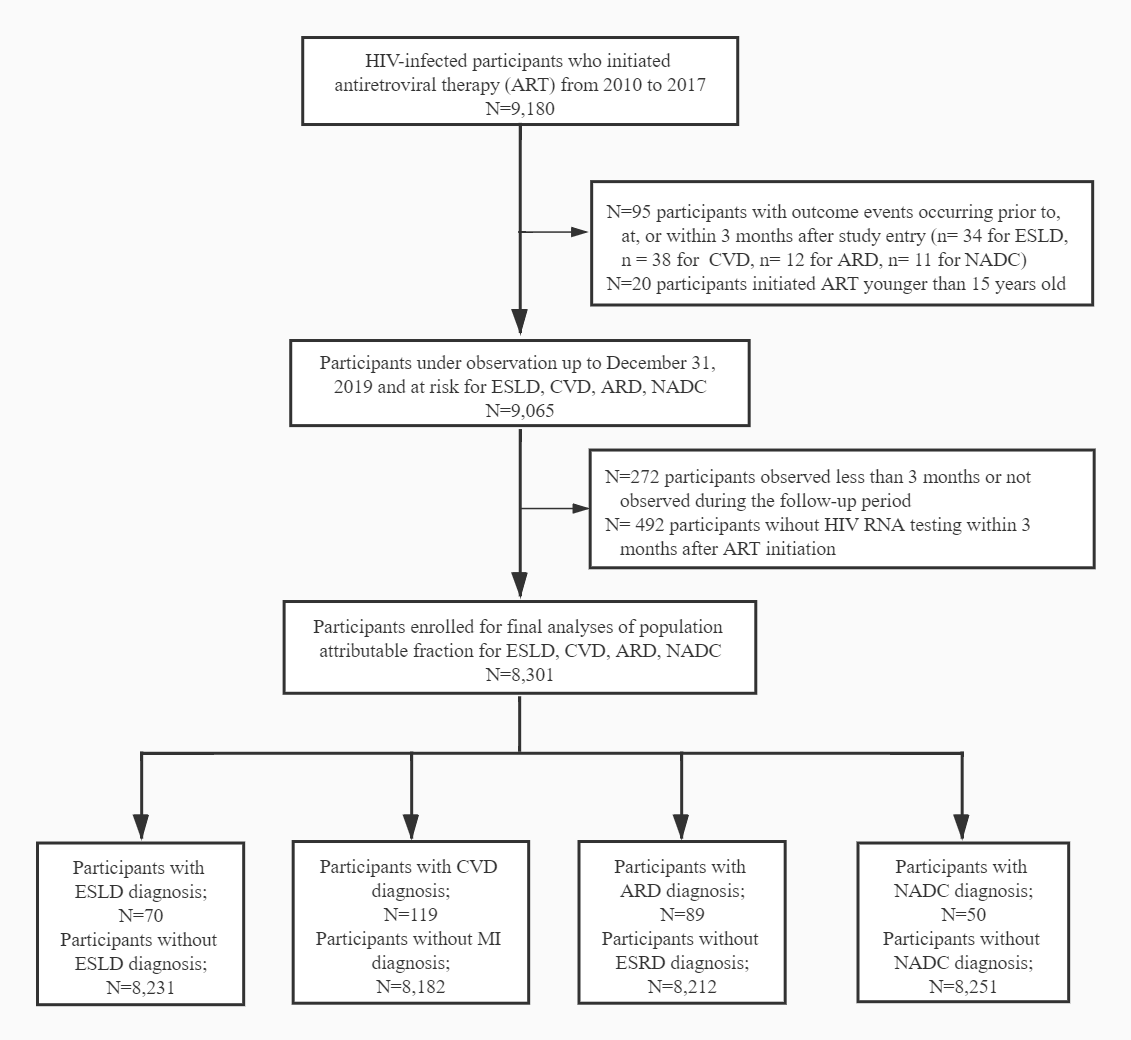


**Supplementary 1 The flowchart of enrolling and excluding participants in this study**

**Supplementary 2 Population attributable fractions of modifiable factors for cardiovascular diseases stratified by age**

| **Risk factors** | **Age (years)** | **PAF (%)** | **95% CI** | **P value** | | |
| --- | --- | --- | --- | --- | --- | --- |
|  |  |  |  | **<30 vs 30-49** | **<30 vs ≥50** | **30-49 vs ≥50** |
| Smoking | <30 | 31.72 | 18.14, 43.04 | 0.9393 | **0.0001** | **< 0.0001** |
|  | 30-49 | 31.77 | 18.76, 42.7 |  |  |  |
|  | ≥50 | 40.17 | 25.83, 51.74 |  |  |  |
| Hypertension | <30 | 2.29 | 0.85, 3.71 | **0.0002** | **0.0001** | **0.0003** |
|  | 30-49 | 5.51 | 2.52, 8.41 |  |  |  |
|  | ≥50 | 15.82 | 7.48, 23.41 |  |  |  |
| Diabetes | <30 | 0.92 | 0.22, 1.62 | **0.0003** | **0.0008** | **0.0019** |
|  | 30-49 | 4.39 | 1.9, 6.82 |  |  |  |
|  | ≥50 | 13.35 | 5.33, 20.69 |  |  |  |
| Hypercholesterolemia | <30 | 1.24 | -3.64, 5.9 | 0.5935 | 0.5695 | 0.5352 |
|  | 30-49 | 1.87 | -5.45, 8.68 |  |  |  |
|  | ≥50 | 2.06 | -5.81, 9.35 |  |  |  |
| Declined eGFR | <30 | -0.44 | -0.83, -0.05 | **0.0374** | 0.0660 | 0.0755 |
| level | 30-49 | -2.71 | -5.26, -0.21 |  |  |  |
|  | ≥50 | -10.95 | -23.17, 0.05 |  |  |  |
| Overweight/Obese | <30 | -3.51 | -8.96, 1.67 | 0.2320 | 0.2958 | 0.8632 |
| (BMI≥ 24 kg/m2) | 30-49 | -5.26 | -13.76, 2.6 |  |  |  |
|  | ≥50 | -5.34 | -14.36, 2.97 |  |  |  |
| HCV infection | <30 | 0.56 | -0.95, 2.04 | 0.4406 | 0.5955 | 0.4541 |
|  | 30-49 | 1.68 | -2.77, 5.93 |  |  |  |
|  | ≥50 | 0.47 | -0.72, 1.65 |  |  |  |
| Low CD4 count | <30 | -0.13 | -11.42, 10.01 | 0.9427 | 0.7288 | 0.3442 |
|  | 30-49 | -0.29 | -16.42, 13.61 |  |  |  |
|  | ≥50 | -1.17 | -19.07, 14.04 |  |  |  |
| HIV RNA ≤500 | <30 | -37.47 | -102.12, 6.5 | 0.2032 | 0.1321 | 0.1100 |
| copies/mL | 30-49 | -33.19 | -88.86, 6.08 |  |  |  |
|  | ≥50 | -21.29 | -58.67, 7.28 |  |  |  |

eGFR=estimated glomerular filtration rate. BMI=body-mass index. HCV=hepatitis C virus.

**Supplementary 3 Population attributable fractions of modifiable factors for cardiovascular diseases stratified by sex**

| **Risk factors** | **Sex** | **PAF (%)** | **95% CI** | **P value** |
| --- | --- | --- | --- | --- |
| Smoking | Male | 38.07 | 23.34, 49.97 | **< 0.0001** |
|  | Female | 8.13 | 3.74, 12.31 |  |
| Hypertension | Male | 7.88 | 3.7, 11.88 | 0.5432 |
|  | Female | 8.51 | 3.87, 12.92 |  |
| Diabetes | Male | 6.38 | 2.72, 9.91 | 0.7958 |
|  | Female | 6.72 | 2.18, 11.06 |  |
| Hypercholesterolemia | Male | 1.72 | -4.94, 7.96 | 0.5982 |
|  | Female | 2.62 | -7.67, 11.92 |  |
| Declined eGFR level | Male | -4.74 | -9.7, 0 | 0.5705 |
|  | Female | -4.52 | -9.29, 0.05 |  |
| Overweight/Obese | Male | -5.16 | -13.58, 2.63 | 0.2306 |
| (BMI≥ 24 kg/m2) | Female | -4.24 | -11.12, 2.21 |  |
| HCV infection | Male | 0.99 | -1.63, 3.53 | 0.4291 |
|  | Female | 2.49 | -4.01, 8.58 |  |
| Low CD4 count | Male | -0.53 | -16.13, 12.97 | 0.9020 |
|  | Female | -0.39 | -18.58, 15.01 |  |
| HIV RNA ≤500 | Male | -30.23 | -81.39, 6.5 | 0.2705 |
| copies/mL | Female | -31.79 | -86, 6.62 |  |

eGFR=estimated glomerular filtration rate. BMI=body-mass index. HCV=hepatitis C virus.

**Supplementary 4 Population attributable fractions of modifiable factors for end-stage liver diseases stratified by age**

| **Risk factors** | **Age (years)** | **PAF (%)** | **95% CI** | **P value** | | |
| --- | --- | --- | --- | --- | --- | --- |
|  |  |  |  | **<30 vs 30-49** | **<30 vs ≥50** | **30-49 vs ≥50** |
| HBV infection | <30 | 22.26 | 6.46, 35.4 | **0.0002** | 0.3258 | **0.0122** |
|  | 30-49 | 26.52 | 8.78, 40.81 |  |  |  |
|  | ≥50 | 20.23 | 5.81, 32.44 |  |  |  |
| HCV infection | <30 | 14.2 | 5.67, 21.95 | **< 0.0001** | 0.3896 | **< 0.0001** |
|  | 30-49 | 33.04 | 16.59, 46.25 |  |  |  |
|  | ≥50 | 16.32 | 6.4, 25.19 |  |  |  |
| Overweight/Obese | <30 | -2.46 | -10.59, 5.07 | 0.5584 | 0.5677 | 0.5918 |
| (BMI≥ 24 kg/m2) | 30-49 | -3.98 | -17.75, 8.17 |  |  |  |
|  | ≥50 | -4.67 | -21.28, 9.66 |  |  |  |
| Alcohol consumption | <30 | -9.77 | -26.08, 4.43 | 0.2162 | 0.7164 | 0.4208 |
|  | 30-49 | -8.58 | -22.81, 3.99 |  |  |  |
|  | ≥50 | -10.25 | -28.67, 5.54 |  |  |  |
| Hypercholesterolemia | <30 | 1.44 | -7.12, 9.32 | 0.7209 | 0.7158 | 0.7139 |
|  | 30-49 | 2.02 | -10.08, 12.79 |  |  |  |
|  | ≥50 | 3.35 | -17.06, 20.21 |  |  |  |
| Low CD4 count | <30 | 10.43 | -13.35, 29.22 | 0.2498 | 0.2939 | 0.6825 |
|  | 30-49 | 14.73 | -18.96, 38.88 |  |  |  |
|  | ≥50 | 15.08 | -20.49, 40.14 |  |  |  |
| HIV RNA ≤500 | <30 | 6.3 | -113.19, 58.82 | 0.9041 | 0.5954 | 0.0413 |
| copies/mL | 30-49 | 6.05 | -104.23, 56.78 |  |  |  |
|  | ≥50 | 7.53 | -103.07, 57.89 |  |  |  |

BMI=body-mass index. HBV=hepatitis B virus. HCV=hepatitis C virus.

**Supplementary 5 Population attributable fractions of modifiable factors for end-stage liver diseases stratified by sex**

| **Risk factors** | **Sex** | **PAF (%)** | **95% CI** | **P value** |
| --- | --- | --- | --- | --- |
| HBV infection | Male | 24.82 | 7.93, 38.61 | **0.0329** |
|  | Female | 21.6 | 6.61, 34.19 |  |
| HCV infection | Male | 22.76 | 10.49, 33.35 | **0.0000** |
|  | Female | 38.91 | 20.7, 52.94 |  |
| Overweight/Obese | Male | -3.65 | -16.2, 7.54 | 0.5948 |
| (BMI≥ 24 kg/m2) | Female | -3.46 | -15.35, 7.2 |  |
| Alcohol consumption | Male | -10.45 | -28.22, 4.85 | 0.2192 |
|  | Female | -1.46 | -3.64, 0.68 |  |
| Hypercholesterolemia | Male | 1.92 | -9.51, 12.16 | 0.7208 |
|  | Female | 2.75 | -13.88, 16.94 |  |
| Low CD4 count | Male | 13.05 | -16.86, 35.31 | 0.2212 |
|  | Female | 16.2 | -20.82, 41.88 |  |
| HIV RNA ≤500 | Male | 6.36 | -106., 57.43 | 0.8702 |
| copies/mL | Female | 6.17 | -110.88, 58.25 |  |

BMI=body-mass index. HBV=hepatitis B virus. HCV=hepatitis C virus.

**Supplementary 6 Population attributable fractions of modifiable factors for advanced renal diseases stratified by age**

| **Risk factors** | **Age (years)** | **PAF (%)** | **95% CI** | **P value** | | |
| --- | --- | --- | --- | --- | --- | --- |
|  |  |  |  | **<30 vs 30-49** | **<30 vs ≥50** | **30-49 vs ≥50** |
| Diabetes | <30 | 0.83 | 0.21, 1.45 | **0.0044** | **< 0.0001** | **< 0.0001** |
|  | 30-49 | 4 | 1.17, 6.74 |  |  |  |
|  | ≥50 | 15.91 | 8.04, 23.11 |  |  |  |
| Declined eGFR | <30 | 7.39 | 3.48, 11.14 | **< 0.0001** | **< 0.0001** | **< 0.0001** |
| level | 30-49 | 26.83 | 15.58, 36.58 |  |  |  |
|  | ≥50 | 52.19 | 36.7, 63.88 |  |  |  |
| Hypertension | <30 | 0.07 | -0.34, 0.48 | 0.7309 | 0.7280 | 0.7271 |
|  | 30-49 | 0.35 | -1.66, 2.32 |  |  |  |
|  | ≥50 | 1.08 | -5.2, 6.99 |  |  |  |
| Smoking | <30 | -1.35 | -10.82, 7.31 | 0.7921 | 0.7937 | 0.7939 |
|  | 30-49 | -1.49 | -12.07, 8.1 |  |  |  |
|  | ≥50 | -2.78 | -24.59, 15.21 |  |  |  |
| Hypercholesterolemia | <30 | -1.67 | -5.84, 2.33 | 0.4445 | 0.4762 | 0.5413 |
|  | 30-49 | -3.16 | -11.35, 4.44 |  |  |  |
|  | ≥50 | -3.84 | -14.4, 5.74 |  |  |  |
| HCV infection | <30 | 0.08 | -2.11, 2.22 | 0.9397 | 0.9468 | 0.9416 |
|  | 30-49 | 0.21 | -5.66, 5.76 |  |  |  |
|  | ≥50 | 0.03 | -0.79, 0.84 |  |  |  |
| Low CD4 count | <30 | 32.8 | 12.54, 48.36 | **0.0000** | **0.0004** | 0.1047 |
|  | 30-49 | 41.11 | 18.2, 57.6 |  |  |  |
|  | ≥50 | 38.66 | 16.84, 54.75 |  |  |  |
| HIV RNA ≤500 | <30 | -49.76 | -122.01, -1.02 | 0.1727 | 0.1242 | 0.1078 |
| copies/mL | 30-49 | -44.83 | -107.35, -1.16 |  |  |  |
|  | ≥50 | -32.29 | -75.05, 0.02 |  |  |  |

eGFR=estimated glomerular filtration rate. HCV=hepatitis C virus.

**Supplementary 7 Population attributable fractions of modifiable factors for advanced renal diseases stratified by sex**

| **Risk factors** | **Sex** | **PAF (%)** | **95% CI** | **P value** |
| --- | --- | --- | --- | --- |
| Diabetes | Male | 11.24 | 5.55, 16.59 | **0.0039** |
|  | Female | 6.47 | 2.66, 10.13 |  |
| Declined eGFR level | Male | 41.21 | 27.79, 52.14 | **0.0030** |
|  | Female | 34.29 | 21.6, 44.92 |  |
| Hypertension | Male | 0.78 | -3.76, 5.13 | 0.7353 |
|  | Female | 0.52 | -2.44, 3.4 |  |
| Smoking | Male | -2.69 | -23.35, 14.52 | 0.7807 |
|  | Female | -0.32 | -2.52, 1.83 |  |
| Hypercholesterolemia | Male | -2.91 | -10.59, 4.22 | 0.4695 |
|  | Female | -5.48 | -20.84, 7.93 |  |
| HCV infection | Male | 0.1 | -2.48, 2.61 | 0.9472 |
|  | Female | 0.17 | -4.76, 4.88 |  |
| Low CD4 count | Male | 40.11 | 17.65, 56.45 | 0.0557 |
|  | Female | 37.82 | 16.4, 53.75 |  |
| HIV RNA ≤500 | Male | -38.31 | -90.49, -0.42 | 0.8427 |
| copies/mL | Female | -38.54 | -91.09, -0.44 |  |

eGFR=estimated glomerular filtration rate. HCV=hepatitis C virus.
